# Celecoxib impairs primary human myoblast proliferation and differentiation independent of cyclooxygenase 2 inhibition

**DOI:** 10.14814/phy2.15481

**Published:** 2022-11-02

**Authors:** Ronald W. Matheny, Alexander L. Kolb, Alyssa V. Geddis, Brandon M. Roberts

**Affiliations:** ^1^ Military Performance Division US Army Research Institute of Environmental Medicine Natick Massachusetts USA; ^2^ Military Operational Medicine Research Program Ft. Detrick Maryland USA

**Keywords:** celecoxib, cyclooxygenase, inflammation, mitochondria, myoblast, non‐steroidal anti‐inflammatory drug (NSAID)

## Abstract

The use of non‐steroidal anti‐inflammatory drugs (NSAIDs) for treatment of musculoskeletal injuries is commonplace in the general, athletic, and military populations. While NSAIDs have been studied in a variety of tissues, the effects of NSAIDs on skeletal muscle have not been fully defined. To address this, we investigated the degree to which the cyclooxygenase (COX)‐2‐selective NSAID celecoxib affects muscle cell proliferation, differentiation, anabolic signaling, and mitochondrial function in primary human skeletal myoblasts and myotubes. Primary muscle cells were treated with celecoxib or NS‐398 (a pharmacological inhibitor of COX‐2) as a control. Celecoxib administration significantly reduced myoblast proliferation, viability, fusion, and myotube area in a dose‐dependent manner, whereas NS‐398 had no effect on any of these outcomes. Celecoxib treatment was also associated with reduced phosphorylation of ribosomal protein S6 in myoblasts, and reduced phosphorylation of AKT, p70S6K, S6, and ERK in myotubes. In contrast, NS‐398 did not alter phosphorylation of these molecules in myoblasts or myotubes. In myoblasts, celecoxib significantly reduced mitochondrial membrane potential and respiration, as evidenced by the decreased citric acid cycle (CAC) intermediates cis‐aconitic acid, alpha‐keto‐glutarate acid, succinate acid, and malic acid. Similar results were observed in myotubes, although celecoxib also reduced pyruvic acid, citric acid, and fumaric acid. NS‐398 did not affect CAC intermediates in myoblasts or myotubes. Together, these data reveal that celecoxib inhibits proliferation, differentiation, intracellular signaling, and mitochondrial function in primary human myoblasts and myotubes independent of its function as a COX‐2 inhibitor.

## Graphical abstract

Non‐steroidal anti‐inflammatory drugs (NSAIDs), including celecoxib, inhibit cyclooxygenase (COX) enzymes to reduce inflammation. Celecoxib is a widely‐consumed NSAID with high selectivity and potency toward COX‐2. We found that celecoxib inhibited proliferation, differentiation, intracellular signaling, and mitochondrial respiration in cultured primary human skeletal muscle cells through a mechanism independent of its COX‐2 inhibitory action. Our findings suggest that celecoxib may possess significant off‐target effects in skeletal muscle.

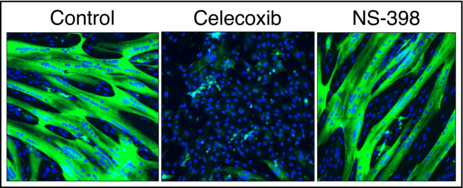

## 1 INTRODUCTION

Physical exercise, musculoskeletal injury, and a variety of illnesses may prompt consumption of pharmaceutical drugs to reduce pain and inflammation (Alemo Munters et al., 2014; Benedetti et al., 2018; Hong & Kim, 2018; Patel & Zwibel, 2020). Non‐steroidal anti‐inflammatory drugs (NSAIDs) possess analgesic and anti‐inflammatory properties that occur by blocking cyclooxygenase (COX) enzymes, thereby inhibiting the production of prostaglandins and decreasing pain and inflammatory responses. In humans, there are two main isoforms of COX proteins (COX‐1 and COX‐2), which are widely distributed across tissues and highly expressed in skeletal muscle (Garcia‐Rayado et al., 2018; Jarrar et al., 2019; Wongrakpanich et al., 2018). COX‐1 is constitutively‐expressed and is involved in gastrointestinal cytoprotection and normal platelet activity, whereas COX‐2 is induced by inflammatory stimuli and regulates inflammation, pain, and fever (Morteau, 2000; Morteau et al., 2000). Prolonged consumption of NSAIDs, particularly those with high potency and selectivity toward COX‐1, may elicit side effects when consumed over long periods; indeed, chronic NSAID use is linked to increased risk of heart attack, stroke, gastrointestinal bleeding, and kidney disease (Garcia‐Rayado et al., 2018; Jarrar et al., 2019; Wongrakpanich et al., 2018). To avoid these side effects, COX‐2‐specific NSAIDs such as celecoxib were developed. However, there is a lack of research regarding the effects of celecoxib on skeletal muscle health.

Understanding the effects of NSAIDs on skeletal muscle health is particularly relevant when considering the increased consumption of NSAIDs in recent decades (Davis et al., 2017). Epidemiological data have shown that NSAID prescriptions and utilizers (those receiving NSAID prescriptions) increased from the year 2006 to 2014 in US Army Soldiers (Walker et al., 2017). Notably, celecoxib was the most‐highly prescribed and utilized COX‐2 inhibitor in this population, with prescriptions of celecoxib increasing more than 3‐fold (from 17,643 prescriptions in 2006 to 57,165 prescriptions in 2014), with a corresponding increase in utilizers. These observations support the need to gain further insight regarding the extent to which NSAIDs in general, and COX‐2 inhibitors in particular, perturb skeletal muscle biology.

Myoblasts and myotubes provide cell‐based models to understand the mechanisms that influence proliferation and differentiation of skeletal muscle. In vivo, myoblasts proliferate, fuse, and differentiate into muscle fibers to restore or increase muscle volume and homeostasis following exercise or muscle injury. As such, myoblasts are critical mediators of skeletal muscle repair, and perturbations to this process increase the risk for inefficient or incomplete skeletal muscle healing. The quality, nature, and type of injury to skeletal muscle is also an important consideration, as previous reports have shown that morphological, physiological, and functional recovery from injury may be regulated through alternative processes (Rathbone et al., 2003; Warren et al., 2007). Previous research indicates that NSAIDs interfere with satellite cell activity (Mikkelsen et al., 2009), translational signaling (Markworth et al., 2014), and protein synthesis (Trappe et al., 2002) in response to a single bout of exercise. However, there have been only limited published reports related to COX‐2 inhibitors, such as celecoxib, and whether these inhibitors affect skeletal muscle growth and regeneration (Bondesen et al., 2006; Paulsen et al., 2010). Given the increased consumption of NSAIDs worldwide and given the importance of muscle cell proliferation and differentiation for skeletal muscle health, we investigated the effects of celecoxib on myoblast proliferation, differentiation, intracellular signaling, and mitochondrial respiration. Our findings indicate that celecoxib significantly inhibits all of these processes through a mechanism independent of its COX‐2 inhibitory function.

## 2 MATERIALS AND METHODS

### 2.1 Materials and reagents

Human Skeletal Myoblasts (Cat #2580), Skeletal Muscle Cell Growth Media and Bullet Kit (Cat #3245), Trypsin and Trypsin Neutralizing Solution Reagent Pack (Cat #5034), and Dulbecco's Modified Eagle Medium:F12 (Cat#12‐719F) were obtained from Lonza Technologies. NSAIDs, including NS‐398 (Cat #S8433) and Celecoxib (Cat #S1261) were purchased from SelleckChem. The MTT proliferation (#30‐1010 K) assay was purchased from ATCC. Antibodies for Vinculin (#13901), PARP (#9542), p‐p70 T389 (#9234), Total p70 (#2708), p‐AKT S473 (#9271), Total AKT (#9272), p‐S6 S235/236 (#4858), p‐S6 S240/244 (#2215), Total S6 (#2217), p‐ERK 42/44 (#9101), Total ERK (#9102), COX1 (#9896), and COX2 (#12282) were purchased from Cell Signaling Technologies. Primers/probes for PCR including *PTGS1* (Hs00377726_m1), *PTGS2* (Hs00153133_m1), *MYH1* (Hs00428600_m1), *MYH2* (Hs00430042_m1), *MYH3* (Hs01074230_m1), *MYH7* (Hs01110632_m1), *MYH8* (Hs00267293) and *GAPDH* (Hs99999905_m1), 6 well tissue culture‐treated dishes (Cat #08–772‐1B), phosphate‐buffered saline (16050130), human skeletal myoblast (large vial Cat #A11440, and small vial Cat #A12555), low glucose Dulbecco's modified eagle medium (Cat #1185092), horse serum (Cat #16050301), collagen‐coated 96‐well dish (Cat #08–773‐10), 12‐well cell culture‐treated dish (Cat #08–772‐29), cell‐counting chamber slides (Cat #C10228), and JC‐1 dye (Cat #T3168) were purchased from Thermofisher Scientific. ELISA kits for PGE2 (Cat #514010) and PGF2α (Cat #516011), Arachidonic Acid (Cat #506321) and the purified COX1 (Cat #17616) and COX2 (Cat #60122) peptides were purchased from Caymen Chemicals Mitoplate S‐1 (Cat #14105), MAS Buffer (Cat #72303), and Redox Dye mix (Cat #74353) were purchased from Biolog. Long IGF‐1 (Cat #I1271) was purchased from Sigma Aldrich. The antibody for Myosin Heavy Chain (Cat #MF‐20) for immunohistochemistry was purchased from Developmental Studies Hybridoma Bank. The antibody for myosin heavy chain (MAB4470) for western immunoblotting was purchased from R&D Systems. Image J was downloaded from the National Institutes of Health website (Schneider et al., 2012).

### 2.2 Cell culture, NSAIDs, and IGF‐1

For proliferative experiments Human Skeletal Myoblasts were grown and expanded in Skeletal Muscle Cell Growth Media and Bullet Kit which contains 10% fetal bovine serum, 2% l‐glutamine, 0.1% epidermal growth factor, 0.1% dexamethasone, and 0.1% gentamicin/amphotericin‐B. Skeletal Muscle Cell Growth Media and Bullet Kit at 37°C and 5% CO_2_. All experiments were performed within six passages of receipt from vendor. Cells were seeded at 1.4 × 10^4^ cells per cm^2^ for all experiments and grown for 48 h in 6 well tissue culture treated dishes. Media was refreshed every 24 h. For differentiation experiments human skeletal myoblasts were differentiated in low glucose Dulbecco's Modified Eagle Medium with 2% horse serum at 37°C and 5% CO_2_. Cells were seeded at 3.8 × 10^6^ cells per cm^2^ for all experiments or human skeletal myoblasts were differentiated in Dulbecco's Modified Eagle Medium:F12 with 2% horse serum, 15 mM HEPES and 3.151 g/L Glucose and L‐Glutamine. Cells were plated and differentiated straight from receipt from the vendor. Media was refreshed every 24 h. Arachidonic acid was diluted in ethanol and cells were exposed, where appropriate, to various doses at a final concentration of 0.1%. Cells were exposed to either Celecoxib or NS‐398 diluted in DMSO at various doses at a final concentration of 0.1%. In all cases control cells were treated at equivalent concentration of DMSO and/or Ethanol. Long IGF‐R3 IGF‐1 was diluted in 0.1 M acetic acid, cells were exposed, where appropriate, to various doses at a final concentration of 0.1%.

### 2.3 ELISAs

Conditioned cell media was collected and prepared for ELISA analysis, performed in quadruplicate, as previously described (Matheny Jr. et al., 2012; Matheny Jr. et al., 2015). The ELISAs were used to measure prostaglandin hormone levels PGE_2_ and PGF_2α_ according to the manufacturer protocol.

### 2.4 Myoblast counting and viability assays

Cell counts were used to assess live and dead myoblasts and were performed using an automated cell counter. Primary human skeletal myoblasts were seeded and allowed to grow for 48‐h on a 12‐well cell culture‐treated dish. Myoblasts were removed using trypsin and trypsin‐neutralizing solution and collected for counting. Cells were stained with Trypan Blue (0.4%) to stain for live cells. Cells were loaded into countess cell‐counting chamber slides and counted on an Invitrogen Countess Automated Cell Counter. Live cells and dead cells were counted. Cell viability was measured using 3‐(4,5‐Dimethylthiazol‐2‐Yl)‐2,5‐Diphenyltetrazolium Bromide (MTT). Human Skeletal Myoblasts were allowed to grow for 48‐h on a collagen‐coated 96‐well cell culture‐treated dish and then the MTT assay was performed according to the manufacturer's instructions.

### 2.5 Confocal microscopy

Cells were fixed and prepared for fluorescent confocal microscopy as previously described using an antibody that recognizes myosin heavy chain (Matheny Jr. et al., 2015). Images were prepared from a Zeiss LSM 700 confocal microscope. Five representative images were taken from each slide. The total number of DAPI were calculated by counting in duplicate. Fusion indices were calculated by counting the number of DAPI contained in myotubes as compared to the total number of DAPI where at least three fused DAPI were considered a myotubes. DAPI was counted in duplicate. Myotube area was processed using NIH Image J 1.60.

### 2.6 Protein extraction and immunoblotting

Cellular protein extraction, Bradford Analysis and Immune Blotting was conducted as previously described (Kolb et al., 2018; Sivandzade et al., 2019). Antibodies including Vinculin, p‐p70 T389, Total p70, p‐AKT S473, Total AKT, p‐S6 S235/236, p‐S6 S240/244, Total S6, p‐ERK 42/44, Total ERK, COX1, COX2, and myosin heavy chain were used. Validation for all antibodies (except COX‐1 and ‐2) included all of the following: (Alemo Munters et al., 2014) ensuring that immunoreactivity is observed at the expected relative molecular mass; (Benedetti et al., 2018) the presence of expected isoforms when an antibody was raised against a sequence contained in those isoforms; (Hong & Kim, 2018) response to stimulus, e.g., increased phosphorylation following exogenous administration of IGF‐I; and, (Patel & Zwibel, 2020) reduced expression (or phosphorylation) following stimulation in cells lacking the target protein, or by inhibiting upstream regulators of the target protein. Purified COX1 or COX2 peptide proteins were used as positive controls for COX1 and COX2 immunoblotting. Densiometric quantification analysis was done using NIH Image J 1.60 (Schneider et al., 2012).

### 2.7 RNA isolation, cDNA synthesis and RT‐PCR

RNA isolation, cDNA synthesis, and RT‐PCR were performed as described previously (Matheny Jr. et al., 2017). Measurements of *PTGS1*, *PTGS2*, *MYH1*, *MYH2*, *MYH3*, *MYH7*, and *MYH8* gene expressions were taken when the threshold of detection exceeded background (CT value) and was calculated using the ‐2^ΔΔCT^ method and normalized to the level of *GAPDH* gene expression.

### 2.8 Mitochondria isolation and function analysis

Mitochondria function was examined using the MitoPlate S‐1 from Biolog, which measures the sensitivity of mitochondria to 22 diverse inhibitors. Human skeletal myoblast cells were expanded and then reseeded at 30,000 cells per well on the MitoPlate S‐1 according to the manufacturer's instructions. The plate was incubated with cells at 37°C and 5% CO_2_ for at least 16 h and then was read on a plate reader using a 595‐wavelength filter. To examine the function of the electron transport chain a membrane sensitive JC‐1 Dye was used. Cells were plated and grown for 48 h in collagen‐coated 96‐ well dishes in growth media at 37°C and 5% CO_2_. Once cells were 80% confluency the cells were rinsed with warm Phosphate Buffered Saline (PBS) and then incubated in 5 μM JC‐1 as described by Kolb et al., (Kolb et al., 2018). Following the 30‐minute incubation, the cells were rinsed with warm PBS and dye excitation was measured using Molecular Devices SpectraMax M5 microplate reader with an excitation wavelength of 488 nm. Emission was measured at both 527 and 590 nm (Kolb et al., 2018; Sivandzade et al., 2019).

### 2.9 Statistics

Data are presented as mean ± SD. Statistical analyses were performed using a Student's t‐test when comparing two experimental conditions, or one way analysis of variance (ANOVA) with a Dunnett's post‐hoc when comparing multiple experimental conditions to a control condition. A two‐way ANOVA with Bonferroni's test post hoc was used to analyze differences in total cell numbers and to analyze MyHC and myogenin expression in Figure 9b. Graph Pad Prism 8.1.2. software was used to perform analyses. A *p*‐value <0.05 was considered significant.

## 3 RESULTS

### 3.1 Celecoxib inhibits prostaglandin production in a dose dependent manner in myoblasts

**FIGURE 1:**
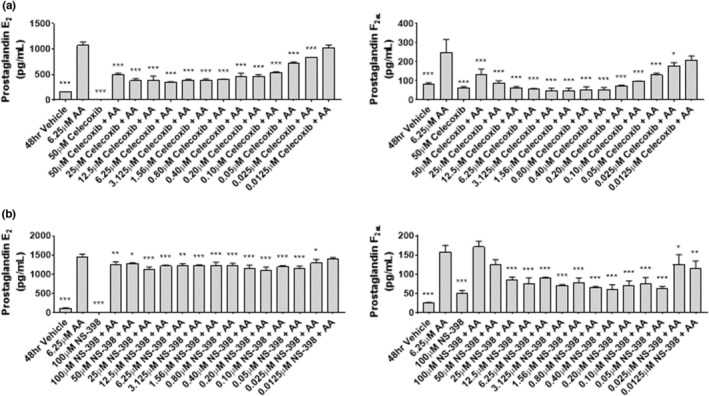
Celecoxib inhibits prostaglandin production in a dose‐dependent manner in primary human myoblasts. (a) Myoblasts were seeded and exposed to the indicated concentration of arachidonic acid (AA), celecoxib, or the combination for 48‐h. Conditioned media was collected and ELISAs performed to determine levels of PGE_2_ or PGF_2_α, as indicated. Data are expressed as mean ± SD and represents four independent determinations (*** *p* < 0.001; * *p* < 0.05). (b) Myoblasts were seeded and exposed to the indicated concentration of arachidonic acid (AA), NS‐398, or the combination for 48‐h. Conditioned media was collected and assayed as described in (a). Data are expressed as mean ± SD and represents four independent determinations (*** *p* < 0.001; ** *p* < 0.01; * *p* < 0.05).

Prostaglandin (PG) synthesis and secretion is a key indicator of COX activity. To determine the efficacy of celecoxib to inhibit PG synthesis in cultured primary human skeletal myoblasts, myoblasts were treated for 48‐h with 6.25 μM arachidonic acid (to induce PGs) in the absence or presence of increasing concentrations of celecoxib (0.0125–50 μM) or NS‐398 (0.0125–100 μM). NS‐398 is a pharmacological inhibitor of COX2 with an IC50 of 0.042 μM, and celecoxib possesses an IC50 of 0.34 toward COX‐2 (Warner et al., 1999). Arachidonic acid stimulated PGE_2_ and PGF_2_α production, while celecoxib prevented the arachidonic acid‐induced PG synthesis in a dose‐dependent fashion (Figure 1a). NS‐398 also inhibited the arachidonic acid‐induced PG response, although to a lesser degree in general (Figure 1b). These data demonstrate that both celecoxib and NS‐398 effectively inhibit arachidonic acid‐induced PG synthesis in primary human myoblasts.

### 3.2 Celecoxib reduces myoblast proliferation in a dose‐dependent manner

**FIGURE 2:**
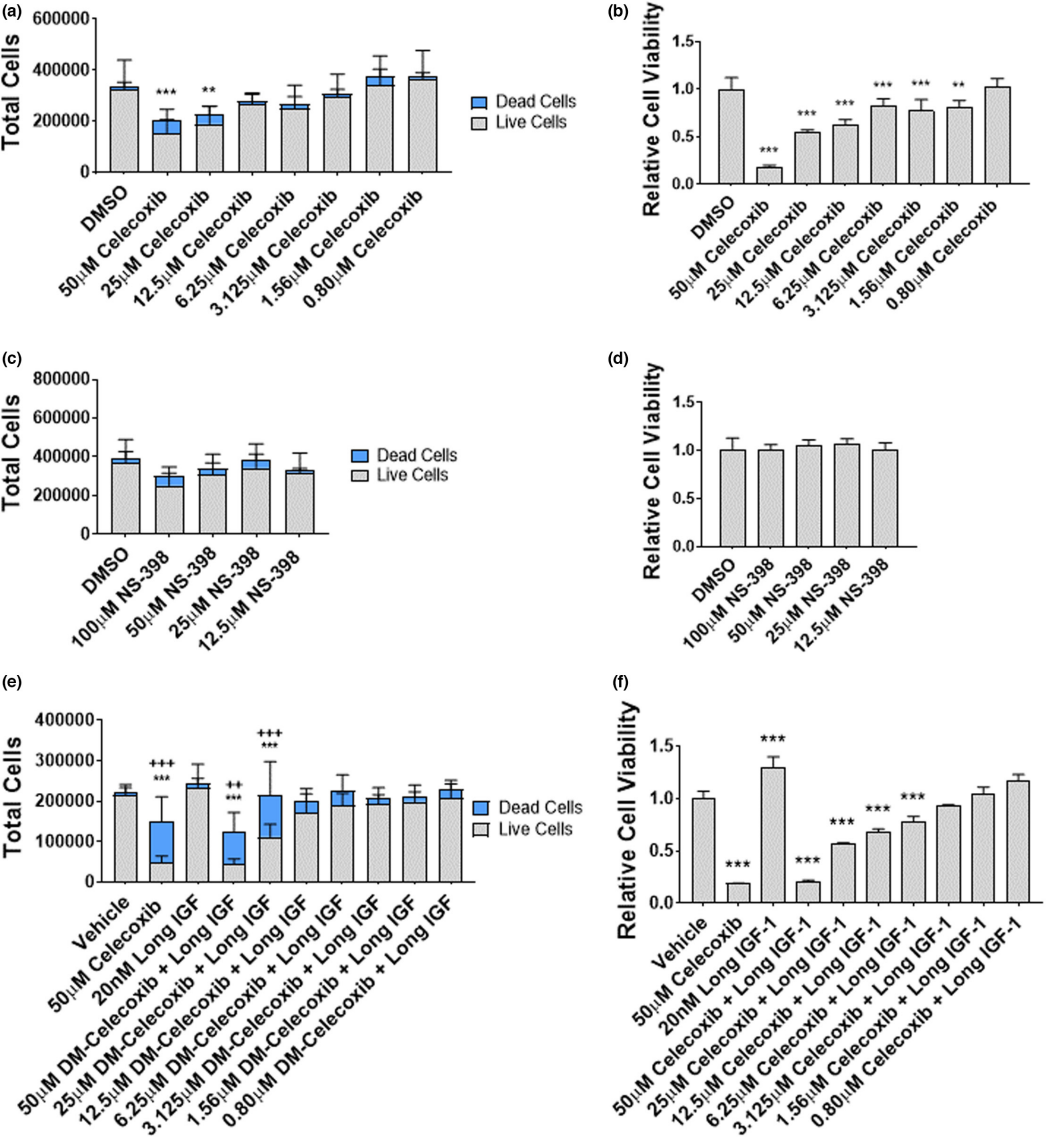
Celecoxib reduces myoblast cell number and viability in a dose‐dependent manner. (a) Myoblasts were seeded and exposed to the indicated concentration of celecoxib for 48‐h. Total cell number was determined using an automated cell counter with live cell staining using trypan blue. Trypan blue‐excluding myoblasts were considered live. Data are expressed as mean ± SD from three independent experiments each performed in triplicate (*** *p* < 0.001; ** *p* < 0.01). (b) Myoblasts were seeded and treated with celecoxib as described in (a), and MTT assay was used to assess cell viability. Data are expressed as mean ± SD from three independent experiments (*** *p* < 0.001; ** *p* < 0.01). (c) Myoblasts were seeded, treated with indicated concentrations of NS‐398, and counted as described in (a). Data are expressed as mean ± SD from three independent experiments, each performed in triplicate. (d) Myoblasts were seeded, treated with indicated concentrations of NS‐398, and MTT assay performed as described in (b). Data are expressed as mean ± SD from three independent experiments, each performed in triplicate. (e) Myoblasts were seeded, treated with indicated concentrations of celecoxib and (or) Long IGF‐I as indicated, and counted as described in (A). Data are expressed as mean ± SD from two independent experiments performed in duplicate (*** *p* < 0.001; ** *p* < 0.01). (f) Myoblasts were seeded, treated with indicated concentrations of celecoxib and (or) Long IGF‐I as indicated, and MTT assay performed as described in (b). Data are expressed as mean ± SD from two independent experiments, each performed in duplicate (*** *p* < 0.001).

We next tested whether celecoxib inhibited myoblast proliferation. Myoblasts were exposed to celecoxib at concentrations ranging from 0.08 to 50 μM for 48‐h, and then counted. Parallel cultures were exposed to NS‐398, at concentrations from 12.5 to 100 μM. 50 μM celecoxib reduced the total number of cells by 53% (*p* < 0.001), and 25 μM of celecoxib reduced the total number of cells by 41% (*p* < 0.01) compared to control (Figure 2a). Celecoxib had a dose dependent effect on myoblast viability as determined by MTT assay, reducing viability by 82% at a concentration of 50 μM (*p* < 0.001), 46% at 25 μM (*p* < 0.001), 38% at 12.5 μM (*p* < 0.001), 17% at 6.25 μM (*p* < 0.001), 23% at 3.125 μM (*p* < 0.001), and 19% at 1.56 μM (*p* < 0.01) (Figure 2b). NS‐398 had no significant effect on myoblast total number, death, or viability at any concentration up to 100 μM (Figure 2c,d).

To determine whether myoblast proliferative capacity could be restored in celecoxib‐treated myoblasts, cells were treated with insulin‐like growth factor‐I (IGF‐I), a peptide hormone known to promote myoblast proliferation and survival. Administration of 20 nM IGF‐I throughout the 48‐hour experimental period was unable to restore the total number of cells in myoblasts treated with 50 μM celecoxib (*p* < 0.001) or 25 μM celecoxib (*p* < 0.001). Furthermore, IGF‐I was not able to restore myoblast viability at any concentration above 3.125 μM (Figure 2f). Similar results were observed when celecoxib‐treated cells were co‐incubated with 200 nM insulin (data not shown). These data demonstrate that celecoxib reduces myoblast proliferation and viability, but that NS‐398 does not, suggesting that the reduced cell numbers and viability observed in celecoxib‐treated cells occurs independent from COX‐2 blockade.

### 3.3 Celecoxib impairs protein synthetic signaling pathways in primary human myoblasts

**FIGURE 3:**
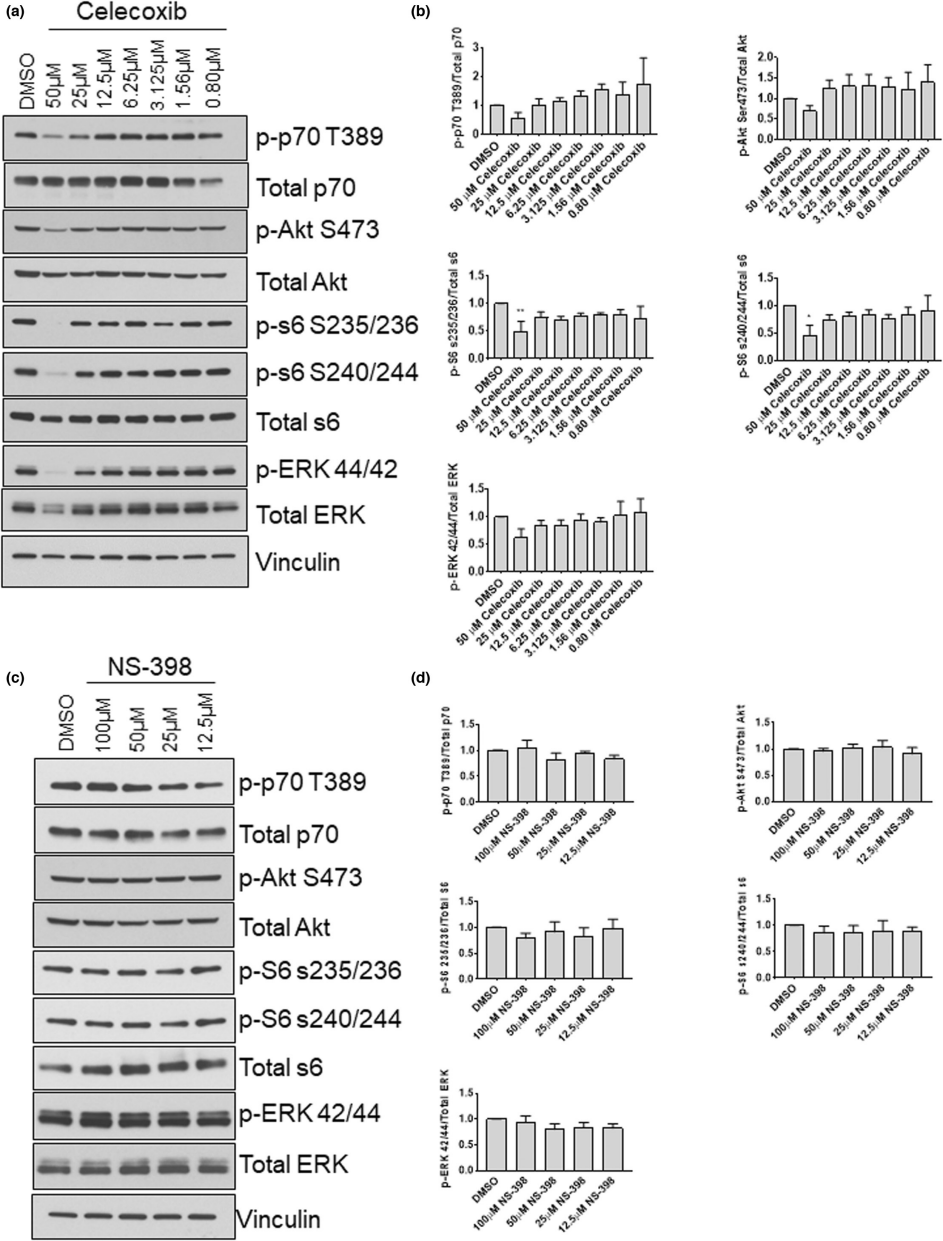
Effects of celecoxib and NS‐398 on anabolic signaling molecules. (a) Myoblasts were treated with the indicated concentration of celecoxib or vehicle (DMSO) for 48‐h. Protein lysates were obtained and Western immunoblotting was performed using the indicated antibodies. (b) Quantifications of blots shown in (a). Phosphorylated protein levels were first normalized to the total expression of the relevant molecule and then expressed relative to DMSO control, which was set at 1.0. Data are expressed as mean ± SD from three independent experiments (** *p* < 0.01; * *p* < 0.05). Myoblasts were treated with the indicated concentration of NS‐398 or vehicle (DMSO) for 48‐h and harvested for protein and Western blot. (d) Quantification of blots shown in (c) and quantified as described in (b).

To determine whether the decreased proliferation in celecoxib‐treated myoblasts was associated with altered intracellular signaling, we examined the expression of several molecules known to contribute to myoblast growth and proliferation. Myoblasts were exposed to 0.08–50 μM celecoxib for 48‐h and harvested. And 50 μM celecoxib significantly decreased phosphorylation of ribosomal protein S6 at Ser 235/236 by 63% (*p* < 0.001), and S6 at Ser 240/244 by 63% (*p* < 0.01) (Figure 3a,b). Nominal, but not significant, decreases in phosphorylated p70S6K (T389), AKT (S473) and ERK (p42/p44) were also observed. NS‐398 had no significant effect on phosphorylation of any of these molecules (Figure 3c,d).

**FIGURE 4:**
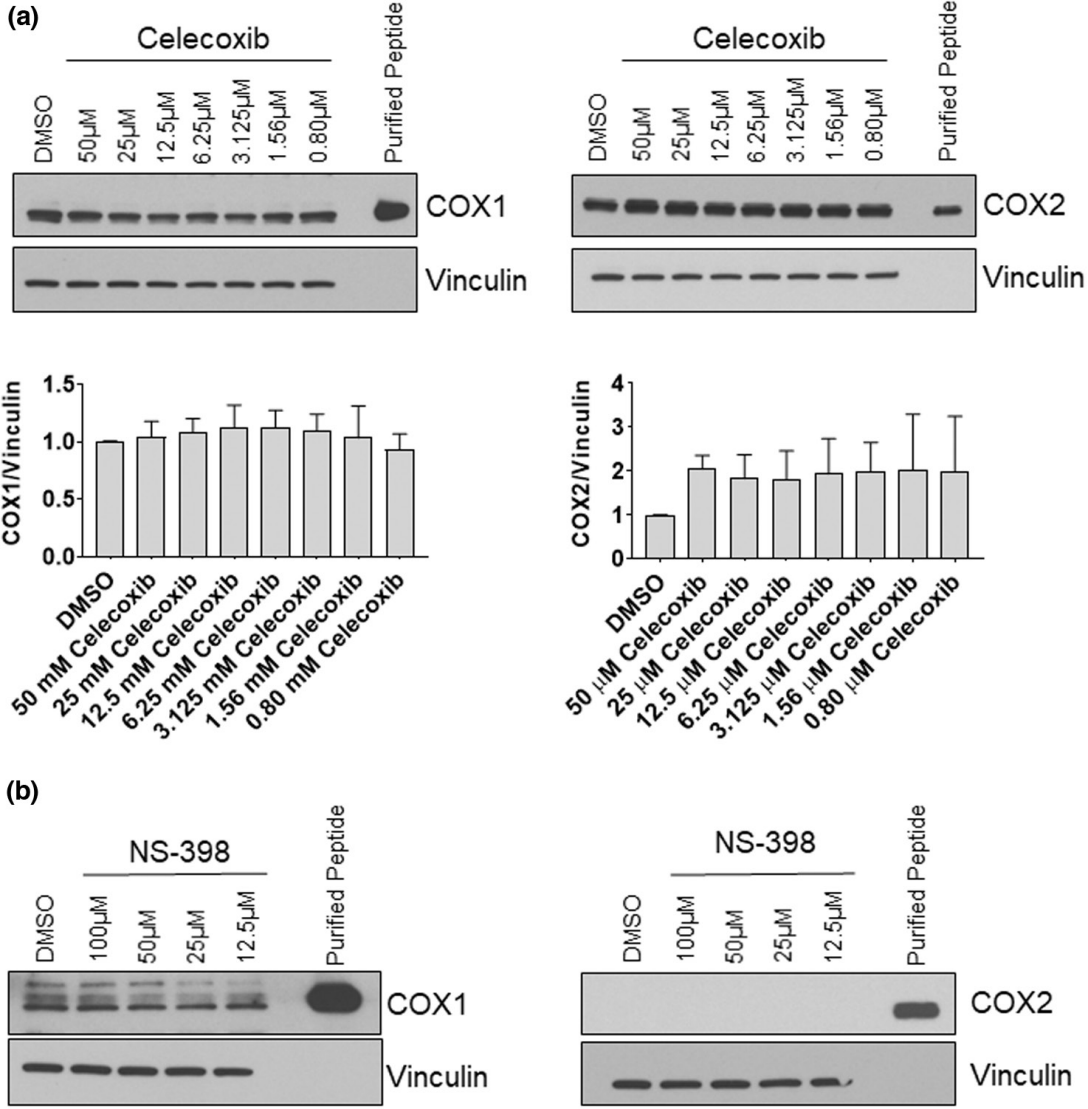
Celecoxib does not alter COX protein in myoblasts. (a) Myoblasts were treated with the indicated concentration of celecoxib or diluent (DMSO) for 48‐h. Protein lysates were obtained and Western immunoblotting was performed using the indicated antibodies. Purified recombinant COX‐1 or COX‐2, identified as “purified peptide,” were subjected to Western blot alongside samples to verify correct antibody immunoreactivity with each isoform. Blots are representative from three independent experiments. Graphs beneath Western blots show quantification of COX‐1 and COX‐2 Western blots. Quantifications were performed by normalizing expression of each COX isoform to vinculin, and then normalized to control which was set at 1.0. (b) Myoblasts were treated and Western blots performed exactly as described in (a) using the indicated concentrations of NS‐398. Blots are representative of three independent experiments.

**FIGURE 5:**
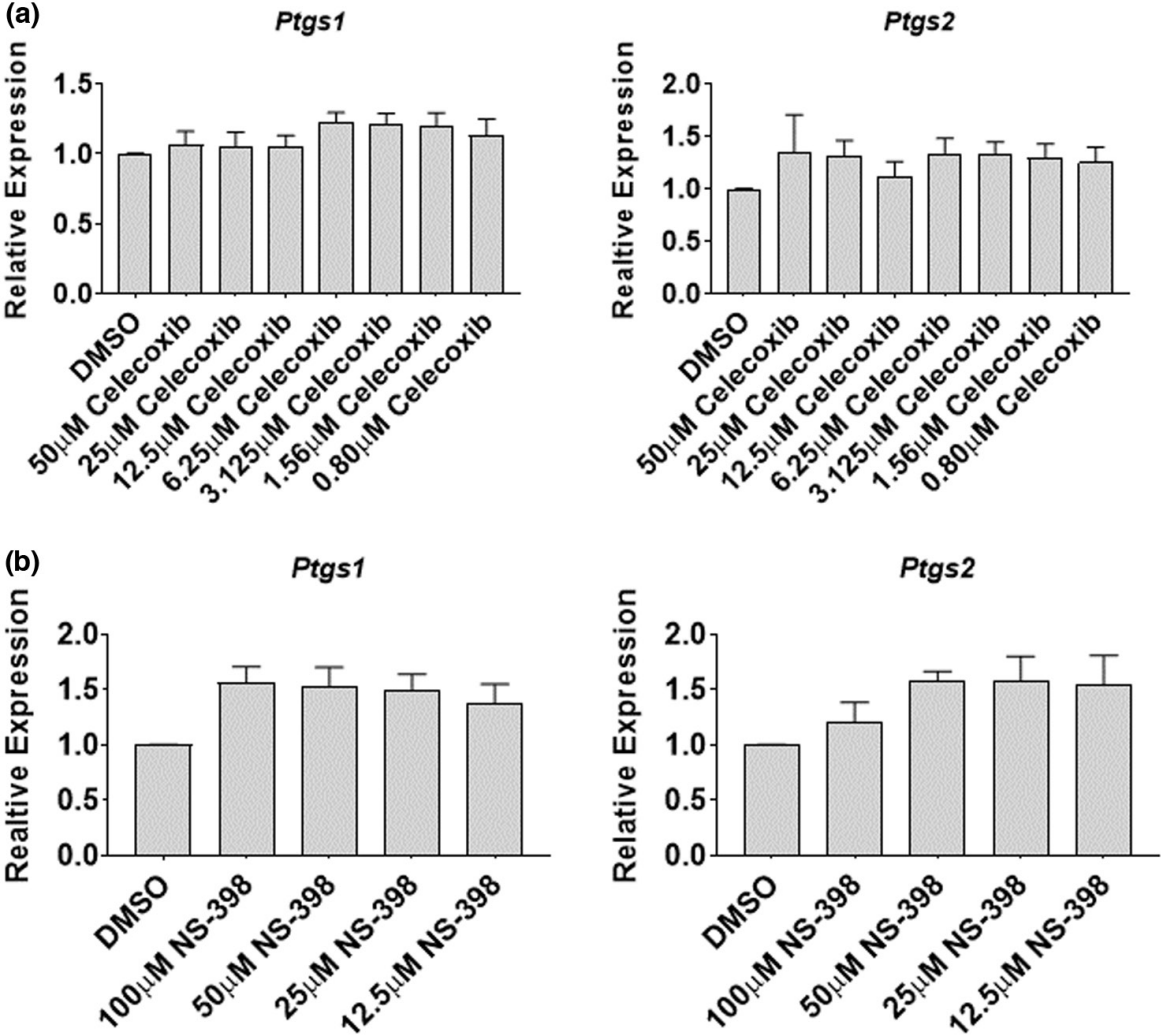
Celecoxib does not affect COX RNA transcript expression in myoblasts. (a) Myoblasts were treated with various concentrations of Celecoxib (a), NS‐398 (b), or vehicle (DMSO) in growth media for 48 h. Cells were harvested 48‐h after seeding, RNA was extracted, and RT‐PCR performed using primers/probes for *PTGS1* (COX‐1) or *PTGS2* (COX‐2) and normalized to *GAPDH* gene expression. Data are expressed as mean ± SD from four independent experiments, each performed in triplicate.

We next investigated whether the altered signaling observed in celecoxib‐treated myoblasts was associated with changes in COX‐1 or COX‐2 transcript or protein levels. Neither celecoxib nor NS‐398 significantly affected levels of COX‐1 or COX‐2 protein (Figure 4) or mRNA (Figure 5) at any concentration tested, suggesting that any cytotoxic effects observed in celecoxib‐treated myoblasts occurred independent of alterations in COX‐1 or COX‐2 transcription or protein abundance.

### 3.4 50 μM Celecoxib reduces mitochondrial membrane potential and respiration in myoblasts

**FIGURE 6:**
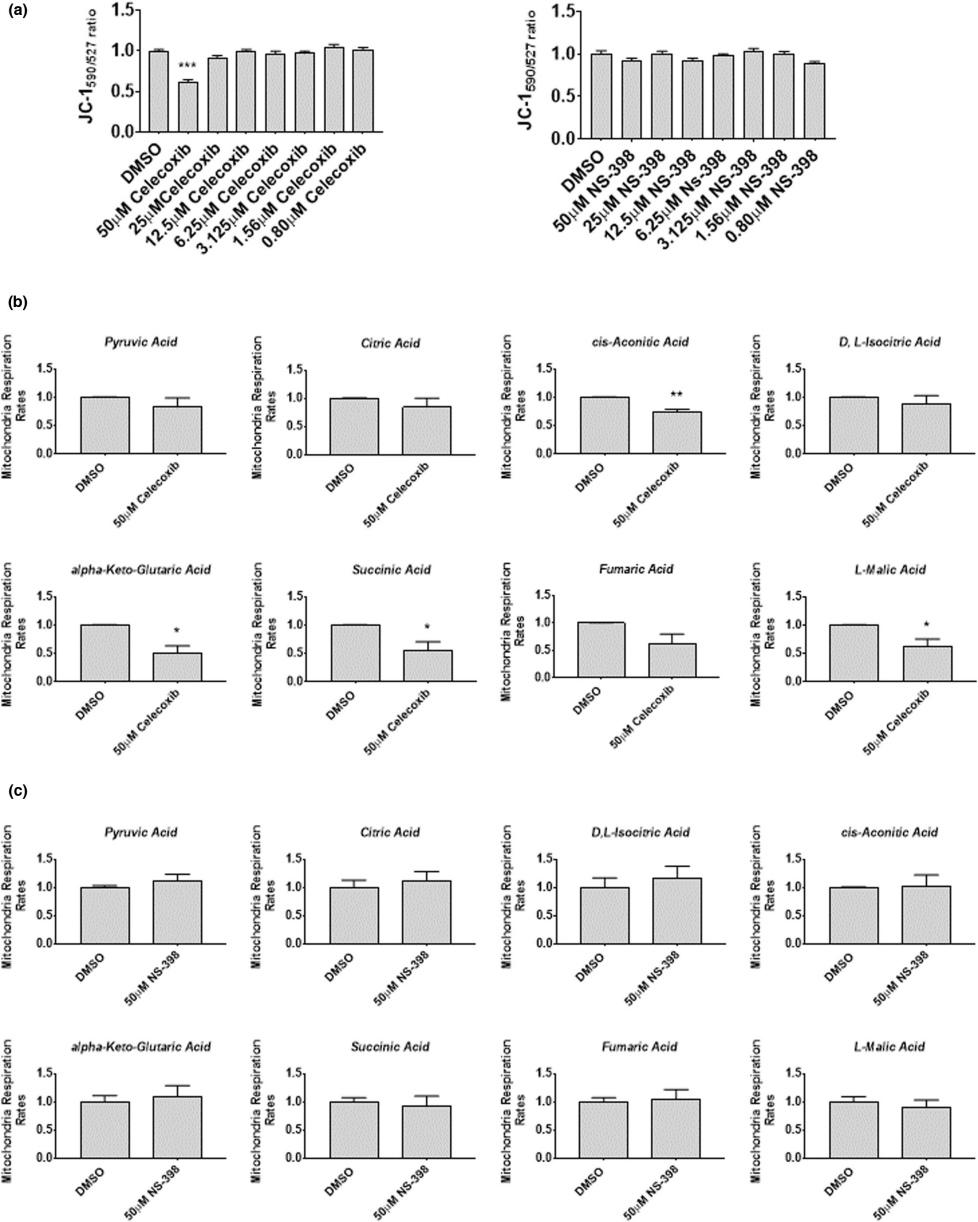
Mitochondrial respiration is reduced in human myoblasts following 48‐h celecoxib treatment. (a) Myoblasts were treated with various concentrations of celecoxib or NS‐398, as indicated, for 48 h. Mitochondria membrane potential was measured using JC‐1, a membrane potential‐sensitive dye. Values from DMSO‐treated control JC‐1590 nm/527 nm ratios were set to 1.0, and all other treatment conditions were expressed relative to DMSO control. Data are expressed as mean ± SD and represent eight experimental replicates. (*** *p* < 0.001). (b) Myoblasts were treated with 50 μM Celecoxib for 48 h. Mitochondrial respiration, using various substrates, was analyzed by measuring the reduction of tetrazolium redox dye as the final electron acceptor. Respiration ratios of DMSO control myoblasts were set to 1.0 for each substrate, and respiration ratios from celecoxib‐treated myoblasts were expressed relative to DMSO control myoblasts. Data are expressed as mean ± SD and represents 3 independent experiments (** *p* < 0.01; * *p* < 0.05). (c) Myoblasts were treated with 50 μM NS‐398 for 48 h. Mitochondrial respiration was assessed and expressed as described in (b). Data are expressed as mean ± SD and represents 3 independent experiments.

Mitochondria are vital for a number of cellular processes including metabolism, growth, and proliferation. To establish whether the decreased proliferation observed in myoblasts exposed to celecoxib was associated with disruptions in mitochondrial function, mitochondrial membrane potential was assessed. Myoblasts treated with 50 μM Celecoxib (Figure 6a) showed a 40% reduction in mitochondria membrane potential compared to cells treated with DMSO vehicle (*p* < 0.001). No changes were observed in cells treated with NS‐398 at any concentration (Figure 6a).

Mitochondrial respiration is critical to muscle cell survival, and the energy equivalents needed to promote respiration are directly linked to changes in membrane potential. To determine whether the reduced membrane potential observed in response to 50 μM Celecoxib was associated with a reduction in mitochondrial respiration, myoblasts were treated with DMSO, 50 μM celecoxib, or 50 μM NS‐398 for 48‐h and citric acid cycle intermediates were measured. Celecoxib‐treated cells possessed reduced activities of the citric acid cycle intermediates cis‐aconitic acid (25%; *p* < 0.01), alpha keto‐glutaric acid (50%; *p* < 0.05), succinic acid (45%; *p* < 0.05), and L‐malic acid (37%; *p* < 0.05) (Figure 6b). Treatment with NS‐398 had no significant effect on the activity of any intermediate (Figure 6c). Taken together, these data suggest that celecoxib‐induced reductions in mitochondrial membrane potential and respiration are not related to COX‐2 inhibition.

### 3.5 Celecoxib inhibits prostaglandin production in a dose‐dependent manner in myotubes

**FIGURE 7:**
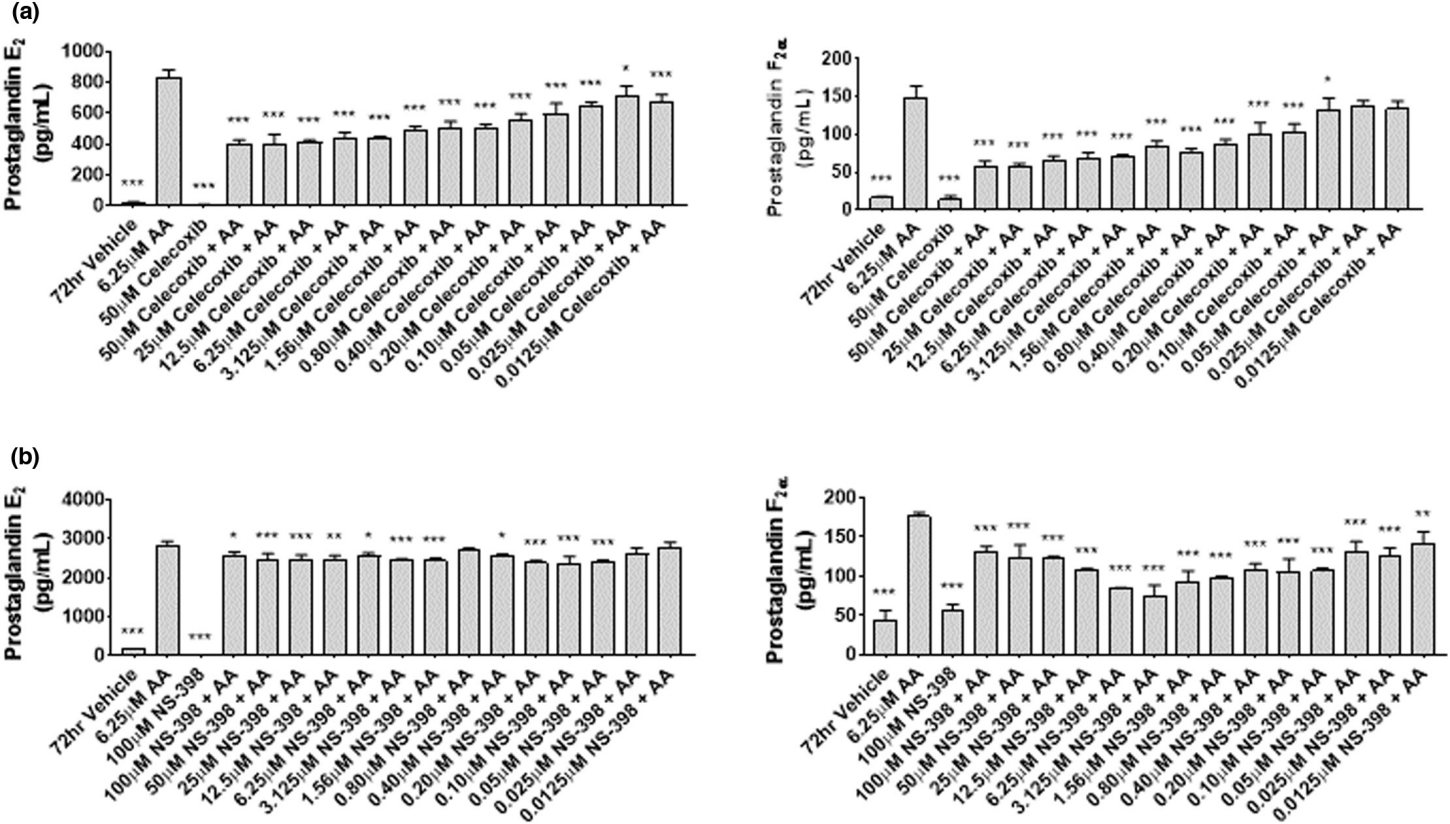
Celecoxib inhibits Prostaglandin production in a dose‐dependent manner in human myotubes. (a) Myoblasts were seeded in differentiation media and exposed to the indicated concentration of arachidonic acid (AA), celecoxib, or the combination for 72‐h. Conditioned media was collected and ELISAs performed to determine levels of PGE_2_ or PGF_2_α, as indicated. Data are expressed as mean ± SD and represents four independent determinations (*** *p* < 0.001; * *p* < 0.05). (b) Myoblasts were seeded and exposed to the indicated concentration of arachidonic acid (AA), NS‐398, or the combination for 48‐h. Conditioned media was collected and assayed as described in (a). Data are expressed as mean ± SD and represents four independent determinations (*** *p* < 0.001; ** *p* < 0.01; * *p* < 0.05).

Myotubes represent the terminally differentiated state of myoblasts that have undergone myogenesis in culture. To determine whether PG synthesis in cultured primary human skeletal myotubes was inhibited by celecoxib to a similar degree as observed in myoblasts, differentiating myoblasts were treated with 6.25 μM arachidonic acid in the absence or presence of increasing concentrations of Celecoxib (0.0125–50 μM) for 72‐h. Similar to myoblasts, arachidonic acid stimulated PGE_2_ and PGF_2_α, while celecoxib prevented the arachidonic acid‐induced PG synthesis in a dose‐dependent fashion (Figure 7a). Differentiating myoblasts treated with NS‐398 also showed inhibition of arachidonic acid‐induced PGE_2_ and PGF_2_α, although to a lesser degree than celecoxib (Figure 7b). These data show that, like in myoblasts, celecoxib and NS‐398 reduce arachidonic acid‐stimulated PG production.

### 3.6 Myotube area, myotube fusion, and myogenic differentiation are reduced with celecoxib treatment

**FIGURE 8:**
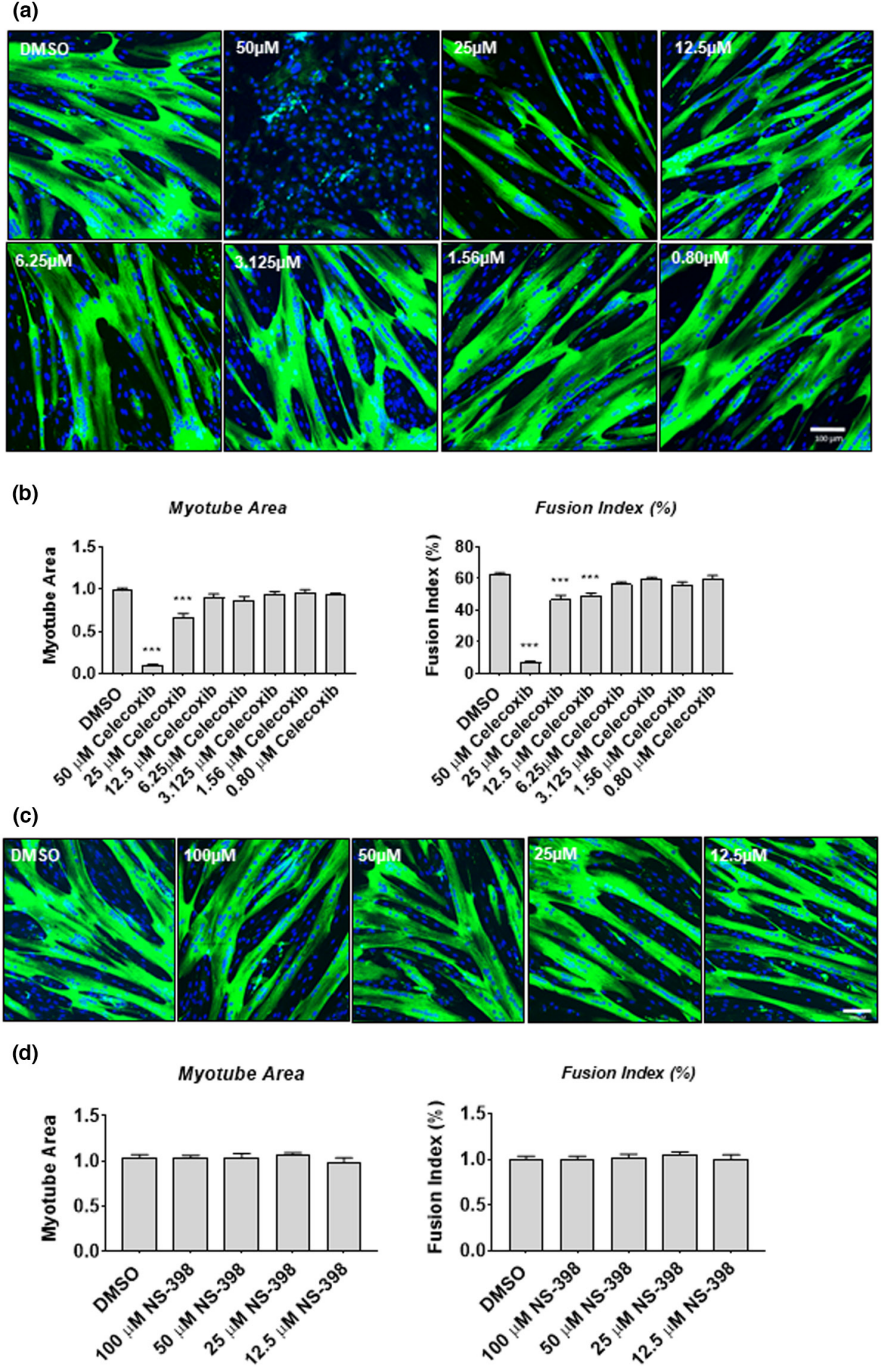
Celecoxib decreases myotube area and fusion index in a dose‐dependent fashion. (a) Myoblasts were exposed to the indicated concentrations of celecoxib or DMSO diluent and allowed to differentiate for 72 h. Cells were fixed and prepared for fluorescent confocal microscopy using an antibody that recognizes embryonic myosin heavy chain (MF‐20). Images were prepared from a Zeiss LSM 700 confocal microscope and processed using Zen software. Celecoxib concentrations are provided in the upper left of each image. (b) Quantification of myotube area and fusion index from experiments shown in (a) (means ± SD; *n* = 3 independent experiments; 5 fields analyzed per experimental point; *** *p* < 0.001). (c) Myoblasts were exposed to the indicated concentrations of NS‐398 (or DMSO) and allowed to differentiate for 72 h. Cells were processed and imaged as described in (a). (d) Quantification of myotube area and fusion index from experiments shown in (c) (means ± SD; *n* = 3 independent experiments; 5 fields analyzed per experimental point).

Given that celecoxib‐reduced proliferation of myoblasts, we next tested whether celecoxib inhibited differentiation by assessing myotube area and fusion. Myotubes were exposed to celecoxib at concentrations ranging from 0.08 to 50 μM for 72‐h (Figure 8a). There was a concentration‐dependent effect of celecoxib on myoblast area, which was reduced by 90% at a concentration of 50 μM (*p* < 0.001) and 34% at 25 μM (*p* < 0.001) (Figure 8b). Similarly, 50 μM celecoxib reduced myotube fusion by 93% (*p* < 0.001), 25 μM of celecoxib‐reduced fusion by 53% (*p* < 0.001), and 12.5 μM of celecoxib‐reduced fusion by 51% (*p* < 0.001) (Figure 8b). We also exposed parallel cultures to NS‐398 at concentrations from 12.5 to 100 μM (Figure 8c), and found that NS‐398 had no significant effect on myotube area or fusion at any concentration tested (Figure 8d). These data reveal that celecoxib, but not NS‐398, impairs myogenic differentiation in a dose‐dependent fashion.

**FIGURE 9:**
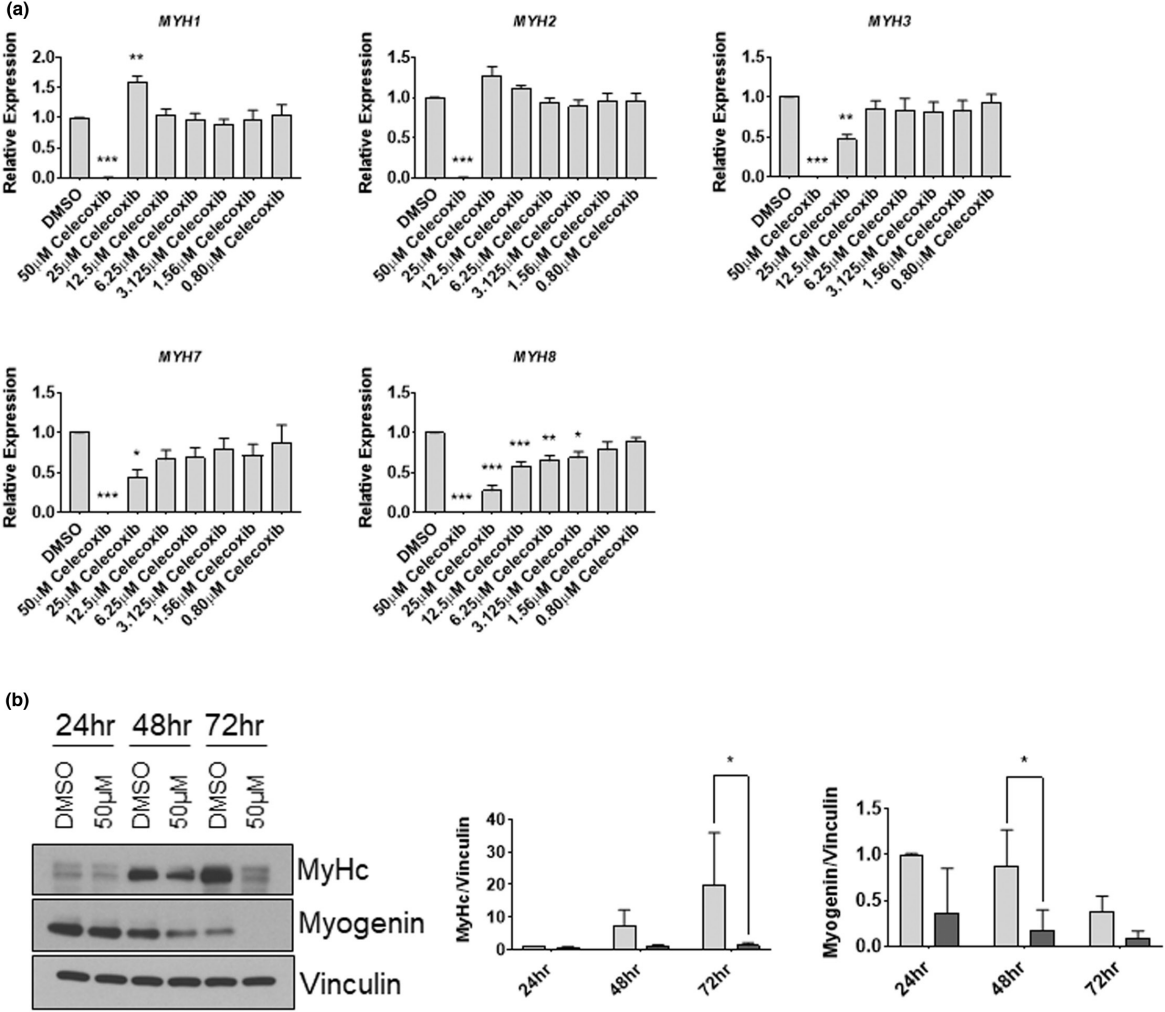
50 μM celecoxib inhibits myosin heavy chain gene expression and myogenin. (a) Myotubes were harvested after 72 h exposure to various concentrations of celecoxib and RNA was extracted. RT‐PCR was performed on cDNA derived from each experimental condition using primers/probes specific for each myosin heavy chain (MYH) isoform. Data are expressed as mean ± SD and represent four independent experiments (*** *p* < 0.001; ** *p* < 0.01; * *p* < 0.05). (b) Myotubes were harvested after a 24, 48, or 72 h exposure to the 50 μM of celecoxib and protein was extracted. Western blotting was performed to evaluate the abundance of myosin heavy chain and myogenin at each indicated time point. Bands from myosin heavy chain and myogenin were expressed relative to the control (DMSO alone) at 24 h, which was set at 1.0. Data are expressed as mean ± SD and represent 3 independent experiments (* *p* < 0.05).

To more deeply examine the phenomenology underlying the celecoxib‐induced reductions in myotube area and fusion, we next investigated the expression of myosin heavy chain isoform RNA transcripts (*MYH1, MYH2, MYH3, MYH7*, and *MYH8*) in response to celecoxib following 72‐h of differentiation. We found that expression of embryonic myosin (*MYH3)* was almost completely eliminated at 50 μM (*p* < 0.001), and reduced by 53% at 25 μM (*p* < 0.01). Similarly, we found slow myosin transcript expression (*MYH7)* were undetectable at 50 μM (*p* < 0.001) and reduced by 56% at 25 μM (*p* < 0.05). Finally, perinatal myosin expression (*MYH8*) was not detected following 50 μM celecoxib treatment (*p* < 0.001), and was reduced by 73% at 25 μM (*p* < 0.001), 43% at 12.5 μM (*p* < 0.001), 35% at 6.25 μM (*p* < 0.01), and 32% at 3.125 μM (*p* < 0.05). Myosin heavy chain IIx/d (*MYH1*) and type IIA myosin heavy chain (*MYH2*) transcripts were both virtually undetectable in cells treated with 50 μM celecoxib, with no reductions in expression of either transcript observed at any concentration below 50 μM (Figure 9a). *MYH1* expression was increased by ~50% (*p* < 0.01) in cells treated with 25 μM celecoxib. These data demonstrate that 50 μM celecoxib is sufficient to almost completely eliminate expression of all MYH transcripts. Notably however, MYH transcripts responded variably to concentrations of celecoxib below 50 μM (ranging from increased expression of *MYH1* following exposure to 25 μM celecoxib, to significantly decreased expression of *MYH8* at 3.125 μM celecoxib), suggesting that each transcript may possess discrete regulatory, expression, or stability characteristics in response to celecoxib.

To identify a mechanism contributing to the substantial lack of differentiation in cells treated with 50 μM celecoxib (Figures 8a and 9a), we next treated cells with 50 μM celecoxib and harvested at 24‐h intervals throughout the 72‐hour differentiation period (Figure 9b). We found that myosin heavy chain, as measured by MF20 antibody, was reduced by 93% at 72 h (*p* < 0.001), consistent with our observations in myotube area and MYH transcript expressions. Myogenin protein was significantly reduced by 80% at 48 h (*p* < 0.05, Figure 9b). The decreased expression of myogenin may explain, at least in part, the observed lack of myosin heavy chain protein (and RNA) because of its role as a muscle‐specific transcription factor regulating myosin heavy chain expression and myogenesis.

### 3.7 Celecoxib impairs protein synthetic‐signaling pathways in primary human myotubes

**FIGURE 10:**
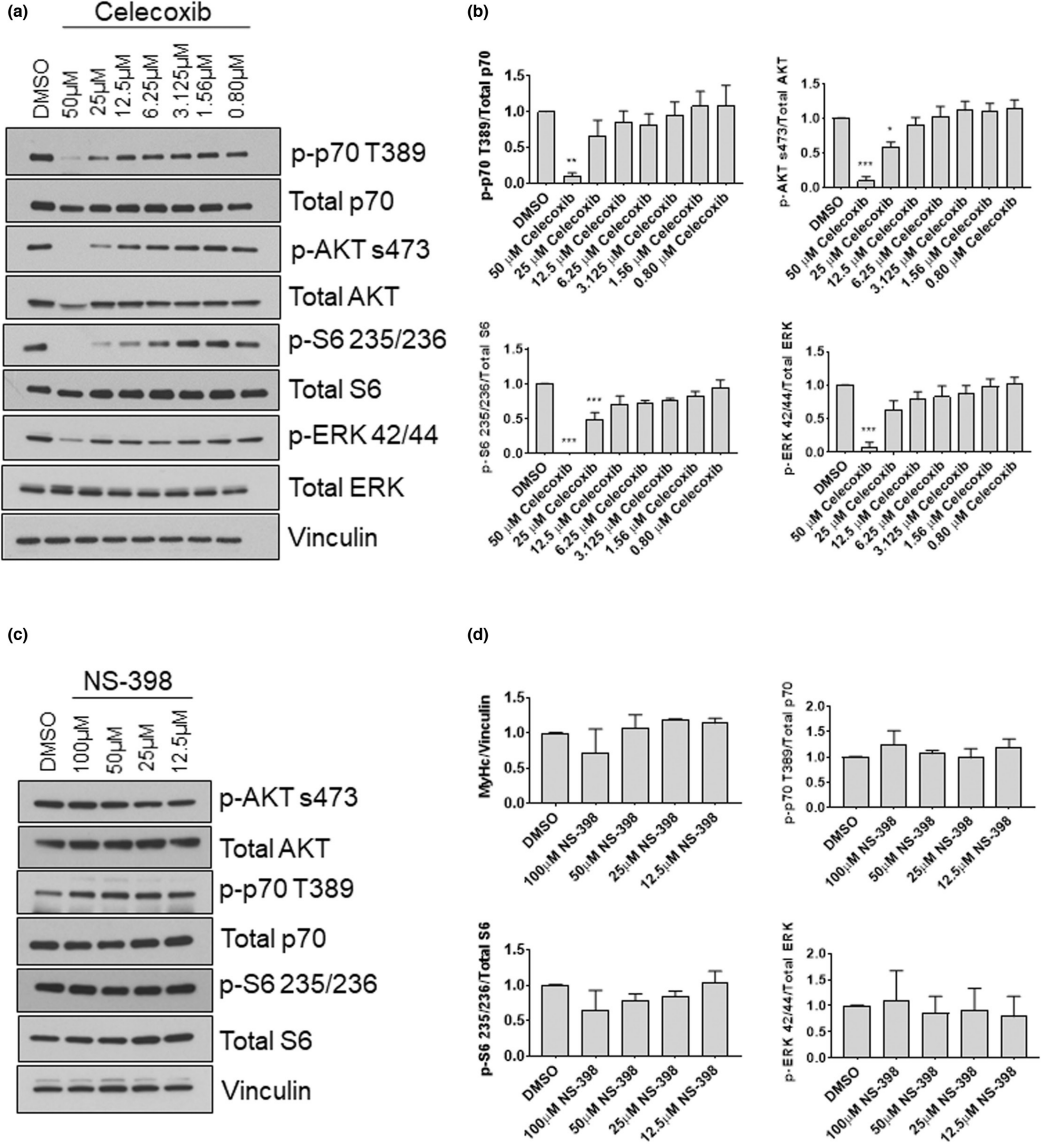
Effects of celecoxib and NS‐398 on signaling molecules involved in myogenic differentiation. Myotubes were harvested after 72 h exposure to the indicated concentrations of celecoxib and protein was extracted. (a) Western blotting was performed to evaluate the abundance of total and phosphorylated p70S6K (T389), AKT (S473), S6 (S235/236) and ERK (42/44). (b) Quantification of blots shown in (a). Bands from phosphorylated p70S6K, AKT, S6, and ERK were first normalized to total expression of these molecules, and then expressed relative to control (DMSO), which was set at 1.0. Data are expressed as mean ± SD and represents 3 independent experiments (*** *p* < 0.001; ** *p* < 0.01; * *p* < 0.05). (c) Western blotting was performed on lysates derived from myotubes exposed to NS‐398 (or DMSO) for 72‐h. (d) Quantifications of blots shown in (c). Data are expressed as mean ± SD and represents 3 independent experiments.

To further explore mechanisms underlying celecoxib‐mediated inhibition of differentiation, we interrogated the AKT and ERK pathways following 72‐h of treatment with 0.8–50 μM celecoxib. We found that p‐p70 T389 was reduced by 90% at 50 μM (*p* < 0.01), p‐AKT S473 was reduced by 91% at 50 μM (*p* < 0.001) and by 42% at 25 μM (*p* < 0.05). Similarly, p‐S6 S235 was reduced by 100% at 50 μM (*p* < 0.001) and by 52% at 25 μM (*p* < 0.05). Furthermore, p‐ERK 42/44 was reduced by 94% at 50 μM (*p* < 0.001) (Figure 10a,b). The abundances of these molecules were unaffected when myotubes were treated with various concentrations of NS‐398 (Figure 10c,d). These data demonstrate that signaling molecules involved in myogenic differentiation are sensitive to celecoxib to a greater extent than observed in myoblasts.

**FIGURE 11:**
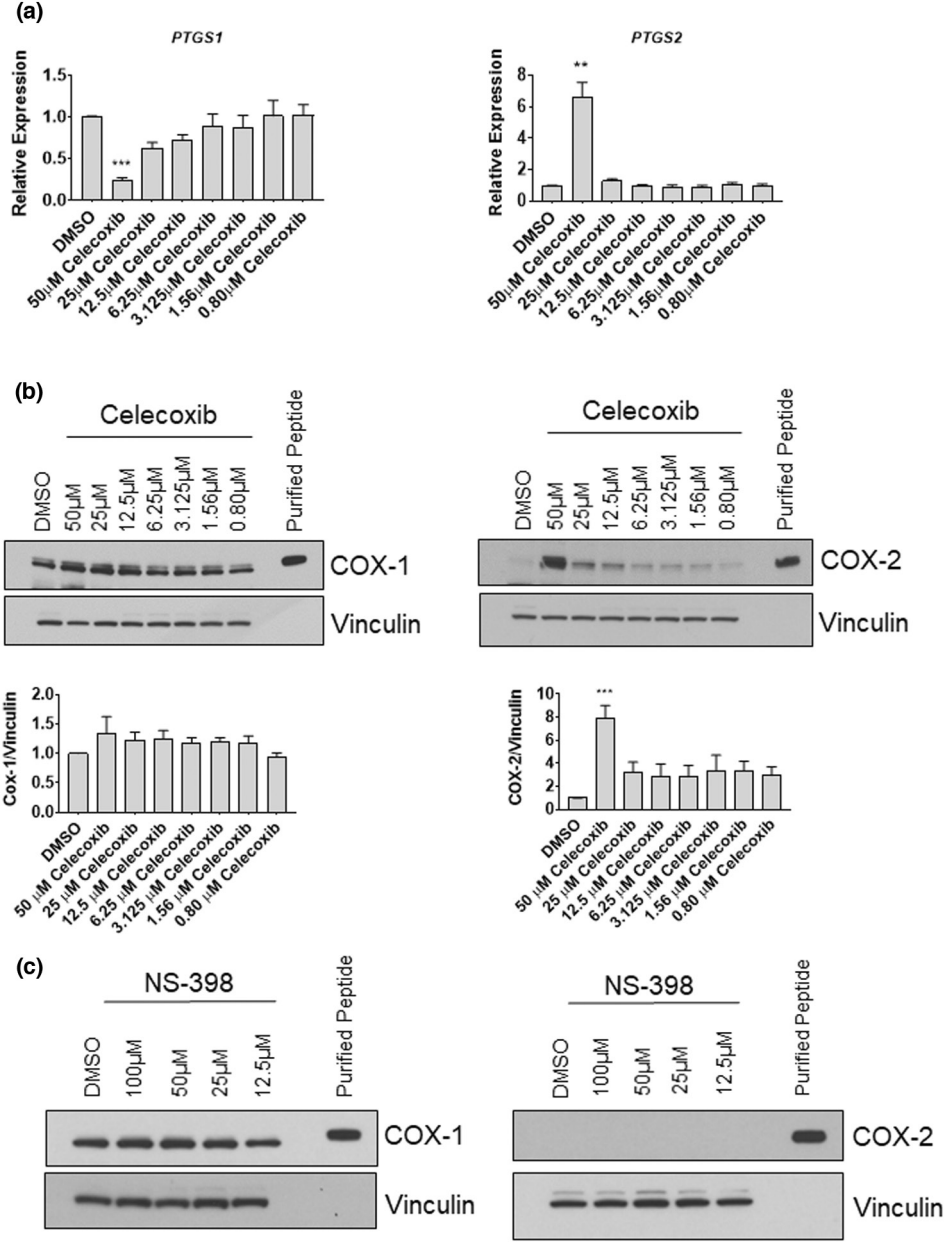
Celecoxib alters COX mRNA, but not protein, in cultured human myotubes. (a) Myoblasts were seeded directly into differentiation media containing the indicated concentrations of celecoxib or diluent control (DMSO). Cells were harvested 72 h after seeding, when differentiation into myotubes was complete. (a) RT‐PCR was performed using primers/probes for *PTGS1* (COX‐1) or *PTGS2* (COX‐2), first normalizing to *GAPDH* gene expression, and then normalizing to DMSO control, which was set to 1.0. Data are expressed as mean ± SD and represent three independent experiments (*p* < 0.001; ** *p* < 0.01). (b) Western blotting was performed on lysates derived from myotubes exposed to the indicated concentrations of celecoxib. Purified recombinant COX‐1 or COX‐2 peptides were electrophoresed alongside experimental samples to verify relative molecular masses of COX enzymes. Data from four independent experiments were quantified as described in Figure legend for Figure 10b, and are expressed as mean ± SD (*** *p* < 0.001). (c) Western blotting was performed on lysates derived from myotubes exposed to NS‐398 (or DMSO) for 72 h. No differences in levels of COX enzymes were observed at any concentration.

Next, we measured COX‐1 and COX‐2 transcript and protein content in response to celecoxib. Unlike our observations in myoblasts (Figures 4 and 5), we found that COX‐1 mRNA (*PTGS1*) was decreased ~74% in cells administered 50 μM celecoxib (*p* < 0.001) (Figure 11a), although there was no difference in COX‐1 protein abundance (Figure 11b). COX‐2 mRNA (*PTGS2*; Figure 11a) and protein (Figure 11b) were increased ~7‐fold and ~ 8‐fold, respectively, when myotubes were treated with 50 μM celecoxib (*p* < 0.001). Consistent with our findings in myoblasts, there were no differences in COX‐1 protein when myotubes were treated with NS‐398 at concentrations up to 100 μM, and COX‐2 was not induced by NS‐398 at any concentration (Figure 11c). These results reveal a unique effect of celecoxib in upregulating COX2 expression in primary human myotubes (but not in primary human myoblasts), suggesting that myotubes possess distinct responses to high concentrations of celecoxib.

### 3.8 50 μM Celecoxib reduces mitochondrial membrane potential and respiration in myotubes

**FIGURE 12:**
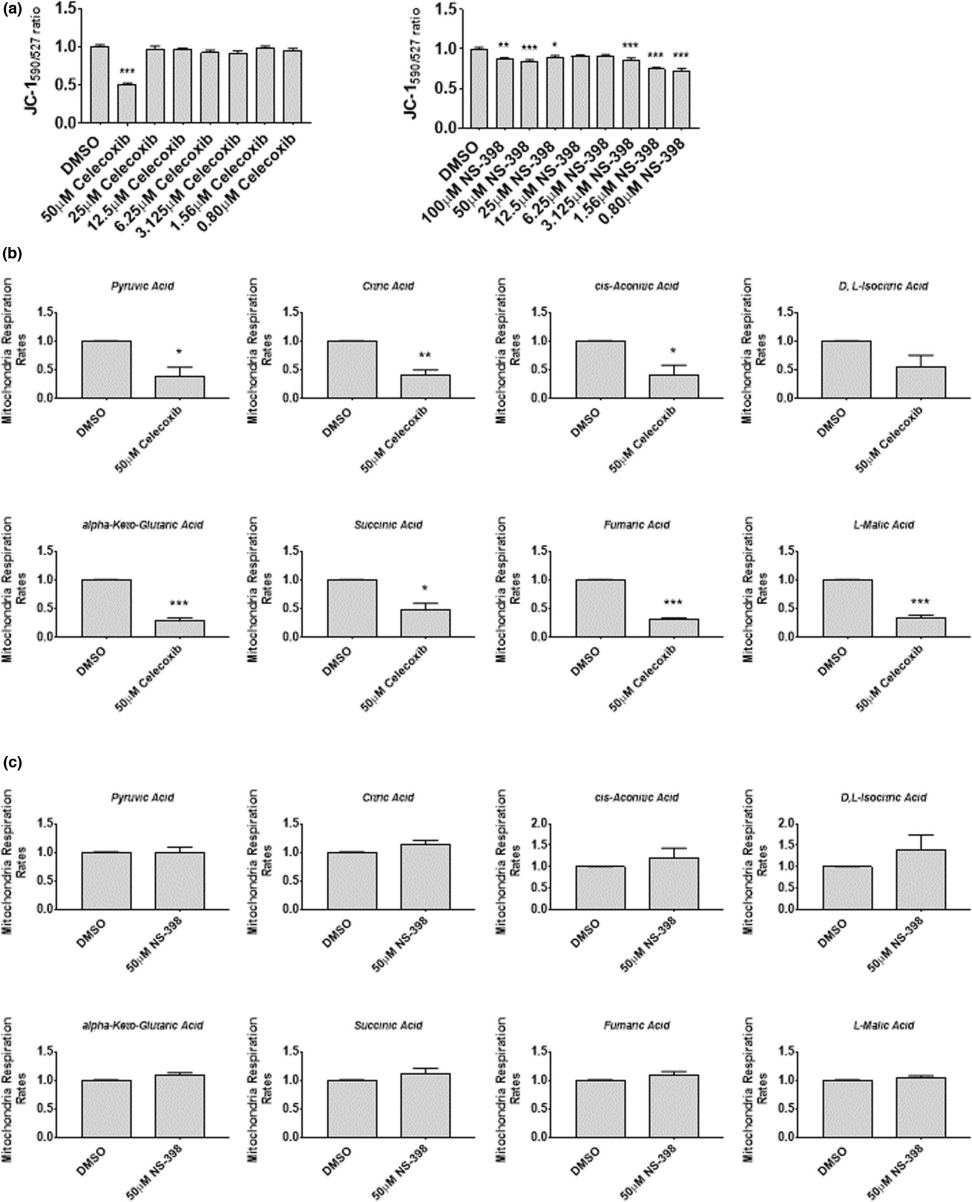
Celecoxib alters mitochondrial membrane potential and respiration in primary human myotubes. (a) Myotubes were treated with various concentrations of Celecoxib or NS‐398 for 72 h. Mitochondria membrane potential was measured using the membrane potential‐sensitive dye JC‐1. Values from DMSO‐treated control JC‐1590 nm/527 nm ratios were set to 1.0, and all other treatment conditions were expressed relative to DMSO control. Data are expressed as mean ± SD and represent eight experimental replicates (*** *p* < 0.001; ** *p* < 0.01; * *p* < 0.05). (b) Myotubes were treated with 50 μM celecoxib in differentiation media for 72 h. Mitochondria respiration, using the indicated substrates, was analyzed by measuring the reduction of tetrazolium redox dye as the final electron acceptor. Respiration ratios of DMSO control myoblasts were set to 1.0 for each substrate, and respiration ratios from celecoxib‐treated myoblasts were expressed relative to DMSO control myoblasts. Data are expressed as mean ± SD and represent 3 independent experiments (*** *p* < 0.001; ** *p* < 0.01; * *p* < 0.05). (c) Myotubes were treated with 50 μM NS‐398 in differentiation media for 72 h. Mitochondria respiration was analyzed using the indicated substrates. Data are expressed as mean ± SD and represent 3 independent experiments.

Given our findings in myoblasts that 50 μM celecoxib reduced mitochondrial membrane potential and respiration, we next examined fully differentiated myotubes. Myotubes were exposed to 50 μM celecoxib (or NS‐398) for 72‐h and harvested for assays. Figure 12a shows that myotubes treated with 50 μM celecoxib showed a significantly reduced membrane potential (~49%) compared to cells treated with DMSO (*p* < 0.001). NS‐398 administration was associated with modest reductions in membrane potential at several doses: 13% at 100 μM (*p* < 0.01), 15% at 50 μM (*p* < 0.001), 11% at 25 μM (*p* < 0.05), 14% at 3.125 μM (*p* < 0.001), 25% at 1.56 μM (*p* < 0.001), and 17% at 0.80 μM (*p* < 0.001).

To determine whether the reduced membrane potentials observed in response to celecoxib or NS‐398 were associated with a reduction in mitochondrial respiration, myotubes were treated with DMSO, 50 μM celecoxib, or 50 μM NS‐398 for 72‐h and citric acid cycle intermediates were measured. Celecoxib significantly reduced pyruvic acid (61%; *p* < 0.05), citric acid (59%; *p* < 0.01), cis‐aconitic acid (58%; *p* < 0.05), alpha‐keto‐glutaric acid (72%; *p* < 0.001), succinic acid (53%; *p* < 0.05), fumaric acid (69%; *p* < 0.001), and L‐malic acid (65%; *p* < 0.001) (Figure 12b). In contrast, we observed no reduction in any citric acid cycle intermediate following treatments with NS‐398 (Figure 12c), suggesting that celecoxib‐induced reductions in mitochondrial respiration are independent of its inhibition of COX‐2.

## 4 DISCUSSION

In this study, we found that celecoxib inhibited proliferation of cultured primary human skeletal myoblasts, attenuated myotube area and myotube fusion, and reduced myosin heavy chain expression and protein content in myotubes. Moreover, celecoxib reduced activation of signaling molecules involved on protein synthesis and differentiation, as well as reduced mitochondrial membrane potential and citric acid cycle intermediates, in both myoblasts and myotubes. None of these effects were observed in cells treated with NS‐398. Taken together, our findings indicate that celecoxib negatively affects myoblast proliferation and differentiation of primary human skeletal muscle cells through a mechanism independent of COX‐2 inhibition.

Myoblast proliferation is important for skeletal muscle development and regenerative capacity. Muscle cells respond to inflammatory stimuli and promote an inflammatory response through prostaglandin (PG) release, which is partly mediated through COX‐dependent enzymatic processes. In muscle cells, the stimulation of PGs, particularly PGE_2_, mediates muscle satellite cell proliferation and supports muscle regeneration (Ho et al., 2017). Additionally, COX‐2‐dependent PG synthesis is essential for muscle regeneration in response to injury (Bondesen et al., 2004), and proliferation of satellite cells (Mendias et al., 2004). While we found that celecoxib reduced myoblast proliferation, myotube growth, and fusion, cells exposed to NS‐398 did not generate the same response. This observation may be partially explained by differences in PGE_2_ and PGF_2α_ activities, receptor abundances, and signaling (Warner et al., 1999); indeed, research suggests that PGE_2_‐mediated intracellular signaling facilitates differentiation, whereas PGF_2α_ facilitates fusion (Ho et al., 2017; Shen et al., 2006). Thus, some of the effects of celecoxib could, at least in part, be due to changes in PGE_2_ and PGF_2α_ absolute and (or) relative concentrations. If this is the case however, celecoxib‐induced reductions in arachidonic acid‐stimulated PG concentrations (which were nominally greater in celecoxib‐treated cells than in NS‐398‐treated cells) likely play only a minor role, as evidenced by the dramatic differences observed in myoblast proliferation, differentiation, signaling, and mitochondrial respiration in celecoxib‐treated cells versus cells treated with NS‐398. Another potential explanation for the negative impacts of celecoxib relates to off‐target effects. High concentrations of celecoxib may affect survival‐ and death‐mediating pathways to favor cell death processes such as apoptosis or necrosis. Such actions may result from the blockade of vital survival signaling pathways, or may result from toxicity of celecoxib itself. This explanation may be at least partially supported by our findings that NS‐398 showed less deleterious effects than celecoxib, even at concentrations up to 100 μM.

To investigate other potential mechanisms underlying the effects of celecoxib, we analyzed signaling pathways known to mediate proliferation, growth, and survival (Garat et al., 2013; Jiang et al., 2017). We found that 50 μM celecoxib significantly reduced phosphorylation of S6 in myoblasts, and phosphorylation of p70S6K, AKT, S6, and ERK in myotubes, suggesting that celecoxib acts, at least in part, through these pathways to impair myoblast proliferation and myotube growth. The reduced S6 phosphorylation following celecoxib treatment, in both myoblasts and myotubes, suggests that translation may be inhibited, which likely contributed to the observed reductions in proliferation and myotube area/hypertrophy (Biever et al., 2015; Dufner & Thomas, 1999; Puighermanal et al., 2017). That myotubes showed reductions in phosphorylation of p70S6K, AKT, and ERK suggests an increased sensitivity to celecoxib in terminally differentiated myotubes compared with myoblasts. In contrast, NS‐398 had no effect on any signaling molecule we examined in myoblasts or myotubes, suggesting that the reduced activation of signaling molecules in celecoxib‐treated cells are independent of celecoxib‐mediated COX‐2 inhibition. It should be noted that some research in animal models suggests these, and other, pathways affected by COX‐2 inhibitors regulate macrophages and neutrophils, which may act on muscle cells in a paracrine manner in vivo. Nonetheless, more research is necessary to understand the exact mechanisms through which celecoxib acts to impair proliferative and myogenic pathways in skeletal muscle cells.

Celecoxib reduced myoblast mitochondrial membrane potential and respiration, suggesting that celecoxib inhibits energy‐dependent processes necessary for myoblast proliferation as well as myotube growth and fusion. While we did not measure ATP directly, changes in respiration and (or) membrane potential offer a proxy for altered ATP production (Suzuki et al., 2018; Zorova et al., 2018). Additionally, our findings revealed substantial and significant reductions in mitochondrial membrane potential and respiration in myoblasts and myotubes exposed to celecoxib. However, when myoblasts were exposed to equimolar concentrations of NS‐398, these differences were not evident. These results suggest that: (1) celecoxib‐induced reductions in myoblast proliferation may be secondary to mitochondrial dysfunction; and, (2) the mitochondrial dysfunction observed in celecoxib‐treated myoblasts likely represents an off‐target effect of celecoxib. In all, celecoxib appears to inhibit myoblast proliferation by impairing multiple energy processes necessary for cell growth and division.

Our study has strengths and limitations. One strength was the employment of a dose–response study design which allowed a systematic examination of concentration‐dependent effects of celecoxib in proliferating and differentiating myoblasts. One limitation of our study concerns the applicability of our findings to tissues other than skeletal muscle, as this study investigated only one cell type (primary human skeletal muscle cells). However, we performed experiments at various stages of the myogenic process ‐ from myoblast proliferation to terminal differentiation ‐ thus covering a comprehensive spectrum of processes in a single cell type. While our results revealed that celecoxib has numerous deleterious effects in primary human skeletal muscle cells, the extent to which these findings may translate to other tissue types is unknown. Finally, we identified several mechanisms through which celecoxib may act to reduce myoblast proliferation and differentiation; however, it is unlikely that we identified all processes or pathways that contribute to these reductions. Future experiments should consider use of omics‐based approaches to determine what pathways may be affected in an unbiased manner.

In summary, we found that celecoxib reduced proliferation, differentiation, anabolic signaling, and mitochondrial function in cultured primary human skeletal myoblasts and myotubes. We identified two primary mechanisms that explain these findings: first, a reduction in pro‐growth and differentiation signaling, including reduced ribosomal protein S6 activation, that may contribute to reductions in protein translation in celecoxib‐treated cells; and second, celecoxib‐induced reductions in mitochondrial membrane potential and respiration that may reduce the energy available for normal metabolic functions, including growth and proliferation. The effects of celecoxib on these processes appear to be independent of its activities on COX‐2, demonstrating that celecoxib can exhibit significant off‐target effects. When placed in a larger context, our findings suggest that celecoxib may exhibit deleterious effects in skeletal muscle apart from its utility as an analgesic or anti‐inflammatory drug, particularly at high doses.

## AUTHOR CONTRIBUTIONS

R. Matheny, A. Kolb, and A. Geddis designed the research; A. Kolb and A. Geddis performed the research; A. Kolb, B. Roberts, and R. Matheny contributed new reagents or analytic tools or analyses; A. Kolb, A. Geddis, B. Roberts, and R. Matheny analyzed the data; A. Kolb, A. Geddis, B. Roberts, and R. Matheny wrote the paper.

## CONFLICTS OF INTEREST

The authors have no conflict of interests to report.

## DISCLAIMER

The opinions or assertions contained herein are the private views of the authors and are not to be construed as official or reflecting the views of the Army or the Department of Defense. Any citations of commercial organizations and trade names in this report do not constitute an official Department of the Army endorsement of approval of the products or services of these organizations.
